# Editorial: Patient safety in low-resource settings: from deficit to capability

**DOI:** 10.3389/frhs.2026.1915407

**Published:** 2026-07-30

**Authors:** Shobhana Nagraj, Eleanor Chatburn, Arthur Kwizera, Tom Bashford

**Affiliations:** 1 Department of Public Health & Primary Care, University of Cambridge, Cambridge, United Kingdom; 2 East London NHS Foundation Trust, London, United Kingdom; 3 Nuffield Department of Women’s & Reproductive Health, University of Oxford, Oxford, United Kingdom; 4 Department of Psychology, Sport and Sensory Sciences, Anglia Ruskin University, Cambridge, United Kingdom; 5 Cambridgeshire and Peterborough NHS Foundation Trust, Cambridge, United Kingdom; 6 Department of Psychology, Centre for Child, Adolescent and Family Research, University of Cambridge, Cambridge, United Kingdom; 7 Department of Anaesthesia and Critical Care, School of Medicine, Makerere University College of Health Sciences, Kampala, Uganda; 8 Department of Engineering, International Health Systems Group, University of Cambridge, Cambridge, United Kingdom; 9 Department of Anaesthesia, Cambridge University Hospitals NHS Foundation Trust, Cambridge, United Kingdom

**Keywords:** implementation science [MeSH], low- and lower-middle-income countries, low resource settings, patient safety, quality of care (QoC), safety culture

Unsafe care is one of the leading causes of avoidable death and disability worldwide, and the burden falls disproportionately on people who receive care in low-resource settings (1). Much of the patient safety literature still treats these contexts primarily through the lens of what is missing: training, equipment, staff, data systems. This Research Topic, ‘Patient Safety in Low Resource Settings’, was assembled to challenge this framing. The articles gathered here span sub-Saharan and West Africa, the Gaza Strip, the WHO European Region, and underserved populations in high-income countries, including the USA. They demonstrate that low-resource settings are also sites of innovation, adaptation, and theory-building from which the global patient safety community has much to learn.

Three major themes connect across the eleven contributions to the special issue. The first is the centrality of culture and reporting systems. Kumah's perspective on adverse event reporting argues that a just culture—one that separates honest error from reckless conduct and that protects those who speak up, is a prerequisite, not an optional add-on for safety improvement in resource-constrained health systems. His companion piece on the future of patient safety in Ghana extends this argument, situating culture alongside leadership, regulation, and education as the foundations on which technical interventions must rest Kumah. Together, these contributions push back against the assumption that safety in low-resource settings is primarily a problem of tools and checklists; it is, first, a problem of the social conditions that make tools and checklists usable in low-resource settings.

The second cross-cutting theme is the implementation of patient safety initiatives in context. Several studies demonstrate that evidence-based interventions do not travel unchanged across settings Kourouma et al., Schuler et al., Gelchu et al. Kourouma and colleagues applied the updated Consolidated Framework for Implementation Research to a modified WHO Safe Childbirth Checklist across three West African countries, highlighting barriers and drivers that would have otherwise been invisible in a purely effectiveness-oriented evaluation Kourouma et al. Schuler and colleagues examined the contextual factors shaping family systems care for small and sick newborns across the care continuum, again showing that fidelity to interventions, without adaptation, is a poor route to safety Schuler et al. Gelchu and colleagues, working in Southern Oromia, Ethiopia, measured health professionals’ readiness for electronic medical record implementation and identified prerequisites that any digital safety intervention must meet before it can be expected to function Gelchu et al. The implication is consistent: implementation science is not a luxury layered on top of patient safety research in low-resource settings; it is an integral need for this research area.

The third theme is the expansion of what counts as a ‘resource’. Edgcombe and colleagues make this argument explicitly, contending that social resources including relationships, trust, communication patterns, and the informal networks that hold fragile systems together are critical and routinely overlooked determinants of safety Edgcombe et al. This insight is reinforced by the article from Abu Rahmah and colleagues on nurses’ compliance with infection control, safety measures, and communication in operating rooms of governmental hospitals in the Gaza Strip, a setting in which conventional measures of resource availability tell only a fraction of the story Abu Rahmah et al. It is reinforced again by Jalei and colleagues’ cross-sectional study of patient satisfaction in two public hospitals in Mogadishu, where patient experience emerges as both an outcome and a feedback signal for safety Jalei et al.. Reframing social and relational capital as resources, rather than as soft accompaniments to ‘real’ resources, opens new and more tractable avenues for improvement.

The collection also brings particular populations into sharper focus. Chatburn and colleagues’ critical review of innovations in community mental health care for children and young people in low- and middle-income countries Chatburn et al., together with Hall and colleagues’ description of the methods underpinning WHO quality standards for child and youth mental health services in the European Region, signal that mental health safety for young people is no longer a peripheral concern, but needs to be embedded into innovations and health services Hall et al.. Rungvivatjarus and colleagues, examining paediatric medication management barriers among providers serving underserved populations, remind us that the analytic category of ‘low-resource’ is not confined to low- and middle- income countries; it describes a relationship between need and available system capacity that can arise anywhere Rungvivatjarus et al.

Together, the articles in this Research Topic suggest a reorientation of the field. They argue for capability-based rather than deficit-based framings of safety in low-resource settings. For example, asking what these systems already do well, often under extraordinary constraint, and how those capabilities can be strengthened and shared. They further argue for methodological pluralism: qualitative implementation studies, perspective and review articles, cross-sectional surveys, and brief reports each carry weight within the special issue, because no single design captures the complexity of safety as a sociotechnical achievement. Lastly, they argue for solidarity across diverse settings. The lessons emerging from Accra, Mogadishu, Gaza, Conakry, Ouagadougou, Bulawayo, and rural Oromia are not local curiosities, they speak directly to safety challenges in better-resourced systems, that have, until recently, mistaken ‘abundance’ for ’safety’.

We are grateful to the authors, reviewers, and Frontiers editorial staff who made this Research Topic possible. The collection is not exhaustive, and surgical safety, antimicrobial stewardship, and the safety of care delivered through community health workers remain under-represented and warrant dedicated future study. We hope, nonetheless, that readers will come away with a clearer view of what low-resource settings contribute to the global patient safety agenda, and a sharper sense of where the next generation of research, policy, and practice should go.
